# Middlesex Hospital

**Published:** 1832-04

**Authors:** 


### HOSPITAL REPORTS.

### MIDDLESEX HOSPITAL.

*Cases of Fever, with Eruption resembling Measles, and early Debility.* By Dr. Wilson.

Case I. George Snow, aged sixty-two, admitted 31st January, with severe pain of head, stupor, slight eruption over the skin, tongue covered with moist fur, surface chilly, pulse not accelerated, though very feeble, bowels confined for three days; says he has a stricture of the rectum, which requires a bougie to be passed before an evacuation is procured. Ill for six days: his indisposition began with chills, but has suffered much from abject poverty. Constant hiccough.—To have Pulv. Jalapæ cum Calomel.; an enema of oatmeal gruel; to be seen by the surgeon.

Died the following day.

*Sectio cadaveris.* The house-surgeon says, there was no stricture of rectum, nor any particular morbid appearance elsewhere.

Case II. George Hervey, age sixty-five, came in the 5th of February, with pain in the head, countenance heavy, abdomen full and rather tense; bowels open; pulse 110, very feeble; general uneasiness. Ill six days; began with severe pain in head, rigors and heats, which have continued ever since.—Spir. Ammon. Aromat. ♏xx. statim. Hydr. cum Cretâ, gr. v. bis die. Cold lotion to head, when hot.

6th. Spots like measles over the body; passes urine and fæces in bed; tongue hard, chapped, black, and dry; low delirium.—Beef-tea, arrowroot; enema of gruel.

Died on the 8th.

These two men were both brought from the same house; and another old man, at the same time, from the same house, and similarly affected, was taken to some other institution, and also died.

Case III. Daniel Dunovan, came in 13th March; aged thirty-five; an Irishman, from St. Giles's: said to have been ill a week, but can obtain no satisfactory account of him. Eyes suffused, lips livid, countenance anxious, extremities cold, pulse scarcely perceptible; says he has pains down the back of his legs; body covered with reddish brown spots.—Warm bath; enema Avenæ; strong broth. Hydr. cum Cretâ, gr. x. bis die.

14th. Has never slept since he came in; delirium constant; starts affrighted; pulse increased since yesterday.—Had four ounces of wine, but no bath.

Died in the afternoon.

*Sectio cadaveris.* Slight reddish blushes at the lower and interior part of ilium; the other abdominal viscera healthy. The larynx slightly red; the trachea more so, as well as the larger bronchia, in which was some frothy mucus; the posterior part of the lungs contained a considerable quantity of blood, attributable to the position after death; structure sound; the anterior portions perfectly healthy. Meninges of brain slightly vascular, and some little serous fluid between the arachnoid and pia mater. Substance of brain, when cut, shewed numerous red spots.

Case IV. A. Thorpe, came in January 31st; age twenty-five, painter: severe pain in head, with deafness and a peculiarity of manner; tongue rosy, chapped, and dry; pulse 100, with power; abdomen soft; says his bowels have not been open for three days. Ill seven days; caught cold, which was followed by febrile indisposition, with spots resembling measles.—The head was shaved, and cold lotion applied. Enema Avenæ daily. Hydr. cum Cretâ, gr. x. bis die.

Feb. 4th. Discharge from left ear.

6th. Mouth sore from medicine. To be left off, and half an ounce of castor oil taken occasionally.

8th. Two tumors below the ears have been opened; and, a few days after, two others, on each side of neck. Discharge has been profuse, and poultices have been continued for about three weeks; debility great, and difficulty of swallowing so much increased as to require the greatest attention of the nurse to support him with wine, arrowroot, and strong beef-tea. Tongue furred, but moist; cannot protrude it, but pulls it out with his hand.—Liq. Morph. Acet. ♏x. at night.

He continued with little alteration for some days, when he began to improve a little; after which, at two different times, a slight erysipelatous blush appeared on the face, so that he could not see without difficulty, and was quite deaf.

On the 25th, had much pain in abdomen, with a red, dry tongue. He had wine, and any other support he wished for. Twelve leeches were applied to the abdomen, although the debility was still great; after which, he continued slowly to improve, and from that time has had no other medicine except half an ounce of castor oil occasionally.

March 17th. Up for the first time; which is a great relief to him, as he has sores on the sacrum and nates. Appetite good.

Case V. Wm. Laurie, age thirty, came in the 30th January: pain in head and abdomen, eyes suffused, no sleep at nights; skin hot, dry, and covered with a faint rubeolous eruption; has been purged; pulse 100, very feeble. Ill six days, with rigors, heats, and followed by pains all over, particularly in head.—Head to be shaved, and, when hot, cold to be applied. Hydr. cum Cretâ et Pulv. Ipecac. co. *aa* gr. v. omni nocte.

31st. Enema Avenæ; twelve leeches to the temples. Pulse, since leeches have been applied, has become scarcely perceptible, but rallied after a dose of Carb. Ammonia.

Feb. 1st. Four ounces of wine daily; arrowroot; beef-tea lb. i.

2d. Calomel. gr. v., and Enema cras mane.

3d. Always delirious; subsultus; spots numerous, and vividly red.—Hydr. cum Cretâ, gr. v. bis die; had a blister to abdomen last night.

4th. Liq. Morph. Acet. ♏x. nocte.

5th. Slept in the night; mouth tender.—Omit the Hydr. cum Cretâ, and repeat the Morph. Acet. at night.

6th. Spots faded; somewhat better.

7th. One ounce of castor oil, which was repeated before the bowels acted.

8th. Subsultus less; tongue rather moist.

10th. Subsultus gone, and spots nearly; tongue clean; slept well; makes no complaint. From which time he continued steadily to improve, and took only some salts or castor oil occasionally.

20th. Half diet.

March 5th. Went out well.

Case VI. Augustus Andrews, admitted February 24th; age twenty: countenance heavy; surface hot, and somewhat moist; pain in head severe, and slight in abdomen, increased on pressure over the liver and iliac region; skin sprinkled with faint but well-marked rubeolous eruptions; bowels opened by medicine; tongue chapped, dry, and white at edges; pulse 100, full. Ill six days: began with rigors, heats, and pain of head; the latter, he says, was relieved greatly by venesection to lb. iss.; has otherwise been worse since.—Apply cold lotion to head, when shaved. Hydr. cum Cretâ, gr. v. ter die.

Went on under this treatment for some days, and saline draughts occasionally: much stupor, very deaf, and a tumor on the left side of neck appeared, which afterwards subsided, without being opened.

On the 5th March, tongue very foul; for which he had Calomel, gr. x.

7th. Tongue cleaner, and slept well last night, after Liq. Morph. Acet. ♏x., Calomel. gr. v. bis.

8th. Bowels open: to have Hydr. cum Cretâ, gr. v. ter die.

10th. Wine, ℥iv. daily; has also beef-tea.

12th. Improving. His tongue has become suddenly swollen to a great degree, dry, red, shining, and impedes respiration.

17th. Tongue nearly natural in size and appearance; still deaf; no complaint. Takes two pints of milk, rice pudding, beef-tea lb. i., and wine oz. iv. daily.

These are some of the cases referred to by Dr. Wilson, in his clinical lecture at the hospital, to shew the peculiarity of the fever which has been in the metropolis for some months past, and which has been marked by the early appearance of debility and protracted recovery.

### *Case of Phlebitis;* by Dr. Wilson.

Ann Durrant, servant, age twenty-three, admitted into the Middlesex Hospital, February 22d: countenance cachectic, sallow, and anxious; surface clammy and pallid; respiration hurried; says she is free from pain; bowels purged by medicine; abdomen full; pulse 140, feeble; tongue tremulous, moist, and pale; cough; no expectoration. Ill three weeks: first had pain of right side, followed by pain in the shoulder, which was relieved by venesection three times, blisters, &c., but made her very weak.—Pulv. Ipecacuanhæ comp., Hydr. cum Cretâ, *aa* gr. v. hăc nocte. Mist. Amygdalæ cum Spir. Æth. Nitrici, ʒss. ter die.

Feb. 24th. Feeble respiration over the superior right side in front, slightly sibilous; œdema of legs; urine slightly acid, not albuminous; great pain in calf of left leg.—Six leeches to the calf, and poppy fomentations.

25th. Pain and swelling of left leg much increased, extending up to the knee, with tenderness in the inside of thigh, but no pain on pressing the groin.—Allowed a pint of porter daily, at her own request.

28th. Cough again troublesome: finds relief from the white mixture; had an opiate last night.

29th. Swelling extended up the whole thigh, with pain now only in the calf; leg and thigh both very large, tense, white, shining, and hot; can only lie on the right side; still no pain in the groin.

March 3d. Died.

*Sectio cadaveris.* The external, internal, and common iliac veins were completely filled with a fibrinous and somewhat white curdy substance, so as to be quite impervious to the circulation; fluid pressed out seemed to be pus and blood mixed: the coats of the veins very much thickened. Peritoneal coat of small intestines injected. The upper and posterior parts of the right lung were marked by *engouement:* the substance of the lung was lacerated with ease, from which exuded, by pressure, a frothy fluid of serum and blood. Had not leave to continue the examination of the vein in the extremity.

